# Parliament and Publications

**Published:** 1911-08-19

**Authors:** 


### PARLIAMENT AND PUBLICATIONS.

### VACCINATION STATISTICS.

In reply to a question asked in the House of Commons this week, Mr. John Burns supplied the following statement giving the number of births registered, the number recorded as successfully vaccinated, and the number of certificates of exemption received in respect of such births :

| Year | Births | Vaccinations | Exemptions |
|---|---|---|---|
| 1903 ... ... | 948,271 | 714,637 | 37,675 |
| 1904 ... ... | 945,389 | 711,504 | 40,461 |
| 1905 ... ... | 929,293 | 705,040 | 44,369 |
| 1906 ... ... | 935,081 | 686,992 | 53 828 |
| 1907 ... ... | 918,042 | 651,050 | 76,709 |
| 1908 ... ... | 940,383 | 594,792 | 160,350 |
| 1909 ... ... | 914,472 | 547,251 | 197,326 |

The large increase in the number of exemptions in the years 1908 and 1909 is somewhat disquieting.

### FREE DISTRIBUTION OF ANTI-TOXIN.

The President of the Local Government Board sees no reason to interfere with the provision for the distribution of anti-toxin, which was made the subject of an official Order about a year ago. When asked in the House of Commons by Mr. Cathcart Wason on Monday this week whether "in view of the fact that the serum treatment of disease is still in the experimental stage, and that the grave dangers of contaminating the blood of human beings with the serum of diseased lower animals is being demonstrated daily, the Local Government Board would consider the advisability of suspending the Order," Mr. Burns replied that the use of anti-toxin in the prevention and treatment of diphtheria is fully justified by medical evidence.

### EXPERIMENTS ON LIVING ANIMALS.

Last year nine new places were registered for the performance of experiments on living animals, and three places were removed from the register. Reports have been furnished to the Home Office by 542 licensees, of whom 147 performed no experiment. The total number of experiments in England and Scotland was 95,731, being 9,454 more than in the previous year. In no instance was a certificate allowed dispensing with the use of anæsthetics for an experiment involving a serious operation; and over ninety thousand of the experiments were performed without anæsthetics, not being of a serious character. Experiments carried out under the provision of the Act requiring that the animal must be kept under an anæsthetic during the whole of the experiment, and must be killed if the pain is likely to continue after the effect of the anæsthetic has ceased, numbered 2,942. The number of experiments performed in the course of cancer investigations was 49,662, being mostly inoculations with mice. In Ireland there were 254 experiments performed by sixteen licensees. "Experiments on Living Animals" (219). Price 6½d.

